# Use of Microfluidic Capillary Electrophoresis for the Determination of Multi-Component Protein Adsorption Isotherms: Application to High-Throughput Analysis for Hydrophobic Interaction Chromatography

**DOI:** 10.3390/pharmaceutics13122135

**Published:** 2021-12-11

**Authors:** Elena Lietta, Alessandro Pieri, Elisa Innocenti, Roberto Pisano, Marco Vanni, Antonello A. Barresi

**Affiliations:** 1 Department of Applied Science and Technology, Politecnico di Torino, 10129 Torino, Italy; elena.lietta@polito.it (E.L.); roberto.pisano@polito.it (R.P.); marco.vanni@polito.it (M.V.); 2 Technological Research and Development, GSK, 53100 Siena, Italy; alessandro.x.pieri@gsk.com (A.P.); elisa.x.innocenti@gsk.com (E.I.)

**Keywords:** high-throughput chromatography, capillary electrophoresis, multi-component adsorption isotherms, hydrophobic interaction chromatography

## Abstract

Chromatography is a widely used separation process for purification of biopharmaceuticals that is able to obtain high purities and concentrations. The phenomena that occur during separation, mass transfer and adsorption are quite complex. To better understand these phenomena and their mechanisms, multi-component adsorption isotherms must be investigated. High-throughput methodologies are a very powerful tool to determine adsorption isotherms and they waste very small amounts of sample and chemicals, but the quantification of component concentrations is a real bottleneck in multi-component isotherm determination. The behavior of bovine serum albumin, *Corynebacterium diphtheriae* CRM_197_ protein and lysozyme, selected as model proteins in binary mixtures with hydrophobic resin, is investigated here. In this work we propose a new method for determining multi-component adsorption isotherms using high-throughput experiments with filter plates, by exploiting microfluidic capillary electrophoresis. The precision and accuracy of the microfluidic capillary electrophoresis platform were evaluated in order to assess the procedure; they were both found to be high and the procedure is thus reliable in determining adsorption isotherms for binary mixtures. Multi-component adsorption isotherms were determined with a totally high-throughput procedure that turned out to be a very fast and powerful tool. The same procedure can be applied to every kind of high-throughput screening.

## 1. Introduction

In the downstream processing of a biopharmaceutical product, chromatography represents the most used technique for the separation and purification of mixtures of proteins [1]. Although chromatography is the most effective and widely used technique to separate a protein, it represents a bottleneck in the downstream processing. It allows us to obtain very high purity and concentration values of the product, but it is a very complex process in which a large number of operating parameters must be taken into account (e.g., type of resin, eluents type and concentration, protein concentration, dimensions of the column, flow rate, etc.) [2].

In the last few years, the development of high-throughput methodologies has allowed the experimental screening of chromatographic systems and operating conditions. Indeed, high-throughput experimental methodologies allow the investigation of several conditions at the same time, using a very low amount of sample and chemicals. For this reason, high-throughput experiments are a very powerful tool to speed up the development of a process while avoiding wasting time and materials [3].

Adsorption thermodynamics have a very important role in the separation efficiency of a chromatographic process. In order to better understand the protein separation in a chromatographic column, adsorption isotherms must be determined [4]. The shape of the adsorption isotherm gives us information about the behavior of a component at the adsorption equilibrium, at a constant temperature. Indeed, the methodology described here is a quick and efficient way to investigate and develop the purification process, since studying adsorption isotherms is propaedeutic to a modeling-based approach, which reduces the experimental effort and optimizes the process.

High-throughput methodology is relatively simple for single component isotherm determination where absorbance analysis is a very quick and precise method for quantification with a throughput that is adequate for the scope. However, in industrial processes the product of interest must be purified from impurities, and to better understand how the adsorption, and then purification of the product is affected by impurities, multi-component isotherms should be investigated. Absorbance analysis is not applicable in this case because to build adsorption isotherms of multi-component systems, quantification of every single component is needed. Thus, analysis of protein mixtures in high-throughput experimentation is still a limitation.

Traditional analytical methods for protein quantification have high resolution and accuracy, but analysis times are quite long and peak integration for quantification is not trivial [5,6] (e.g., HPLC or UPLC). A combination of UV spectrophotometry and multivariate data analysis has been used to reduce the time for quantification, but a high level of statistical knowledge is required to build, calibrate and validate partial least squares (PLS) regression models. Furthermore, bad model prediction can occur if a protein has a low impact on the spectrum with respect to the others in the mixture or if the concentrations are too low and they cannot be isolated from the default noise [7].

In this work, we propose the application of a different analytical method to determine multi-component adsorption isotherms, which are a powerful tool to better understand process dynamics, especially in the process development step. The analytical method exploits microfluidic capillary electrophoresis in a high-throughput platform. This analytical methodology coupled with high-throughput batch experiments in 96-wells filter plates obtains adsorption isotherms in a very fast and easy way. The LabChip high-throughput platform has been successfully validated in a previous work [8] as an analytical method for monoclonal antibodies and as robust and suitable for routine analysis.

The objective of this study was to verify the performance of capillary electrophoresis as a tool for protein quantification in adsorption isotherm determination. The uncertainty of the analytical method was evaluated in terms of precision, accuracy, and separation efficiency when it was exploited for multi-component adsorption isotherm determination. Furthermore, the error that occurs during experimental high-throughput methodology and is due to manipulation was calculated. This error allowed us to evaluate the overall precision of the complete procedure. The experimental error was compared with the UV-Vis analysis in the case of single-component adsorption isotherms. In the case of protein mixtures, the comparison was done with results from similar works found in the literature.

This work can also be considered for comparison with possible new techniques that could be proposed in the future. Lastly, the influence of the manipulation in the high-throughput procedure was investigated in order to assess how it affects the results.

As case studies, the adsorption behavior of three model proteins (BSA, CRM_197_ and lysozyme) in hydrophobic interaction resin was studied. These proteins were chosen because they have different behavior in hydrophobic resin [9] and different molecular weights. The adsorption isotherms of the three proteins as a single component were determined first. Moreover, the overall experimental error was quantified to evaluate the reproducibility of the high-throughput procedure investigating a single condition several times. The proteins’ adsorption behavior was then investigated as binary mixtures. To do this, microfluidic capillary electrophoresis was employed to quantify the protein concentration. As mentioned above, to assess the potential of this procedure, capillary electrophoresis was evaluated under different aspects. The reproducibility of the whole high-throughput procedure (adsorption and analysis) was evaluated for binary mixtures. Then, the accuracy of the high-throughput analysis platform was determined by analyzing a sample with a known concentration. In the end, since the three proteins have different molecular weights, the separation efficiency was rated.

## 2. Materials and Methods

### 2.1. Materials

Bovine serum albumin (BSA) from bovine serum and lysozyme from chicken egg white are purchased from Sigma Aldrich (St. Louis, MO, USA), CRM_197_ was available (stored at −20 °C) from GSK Laboratories (Siena, Italy). The molecular weight of the proteins considered was 66.4 kDa, 14.4 kDa and 58.8 kDa, respectively. CRM_197_ is a non-toxic mutant of diphtheria toxin used as a carrier protein [10]. Potassium monobasic phosphate was purchased from Sigma Aldrich (St. Louis, MO, USA) and ammonium sulfate was from Carlo Erba Reagents (Milan, Italy). Potassium hydroxide used for buffer titration was also purchased from Carlo Erba Reagents. Syringe filters used to filter protein solutions before loading were purchased from Sartorius (Gottongen, Germany). The Butyl Sepharose HP resin bulk was purchased from GE Healthcare (Uppsala, Sweden); the resin has a degree of substitution of 40 μmol butyl groups/mL medium and a mean bed diameter of 34 μm. AcroPrep 96 Filter Plates with PTFE membrane were used for the high-throughput isotherms determination and were purchased from Pall Corporation (New York, NY, USA). Absorbance analysis were performed with a Multiscan Sky Spectrophotometer from Thermo Scientific (Waltham, MA, USA). Analysis of the protein mixtures was performed with the LabChip GXII Touch protein characterization system; the Protein Express Assay LabChip and the Protein Express Assay Reagent Kit were purchased from PerkinElmer (Waltham, MA, USA). Other materials used for LabChip analysis are described in Section 2.4.2.

### 2.2. Procedures and Equipment

Single-component and binary mixture solutions were investigated for this study. In both cases, a stock solution of each protein was prepared to have a protein concentration of 10 mg/mL and filtered using a Minisart Syringe Filter with a cut-off of 0.22 μm. For the mono-component isotherms determination, four solutions with a concentration of 1, 2, 3, 5 mg/mL were prepared by dilution of the stock. Instead, for binary mixtures, four total protein concentrations were investigated: 2, 5, 7, and 10 mg/mL were obtained by mixing and then diluting single protein stock solutions. For each binary mixture, three different protein ratios were considered: 70–30%, 50–50%, and 30–70%. In all cases, the solutions were prepared at three different ammonium sulfate concentrations: 0, 0.5, 1 mol/L in a 0.05 mol/L of potassium monobasic phosphate buffer. A 6 mol/L potassium hydroxide solution was used for titration to reach the neutral pH of the solutions.

The resin bulk was purchased as a slurry of 70% solid phase and 30% storage solution (GE Healthcare, Uppsala, Sweden), which is ethanol 20% v/v. The purchased slurry was allowed to settle in a graduated cylinder, and after the solid phase was settled, water was added to the cylinder to obtain a clear liquid volume equal to the apparent solid volume. The obtained slurry was then maintained under stirring to be loaded in the plate. A vacuum manifold system was used (Pall Corporation, New York, NY, USA) to remove the supernatant from the filter plate in the case of single-component isotherm determination. In the case of binary mixtures, the supernatant was removed by centrifuging (to minimize contamination between different wells).

At first, the wells of the plate were filled with 50 μL of resin slurry, then the resin was washed twice by pipetting 300 μL of water and removing the supernatant. Each aliquot of resin was equilibrated twice with 300 μL of the buffer with the defined salt concentration, then the supernatant was removed. Each well was filled with 120 μL of loading solution (the salt concentration in the loading solution must be the same as the equilibration buffer for each well). The plate was kept under stirring for 6 h at 1300 rpm and 25 °C to be sure that the equilibrium was reached in each well homogeneously. The supernatant was removed from the wells and collected in the collection plates by filtration or centrifugation, as described above.

The collected supernatant and loading solutions were analyzed with a UV spectrophotometer to get the protein concentration values (see Section 2.4.1) for single-component isotherms. Each data set was repeated four times and the average values of free and adsorbed protein concentration were considered. In order to prove the consistency of LabChip analysis, single component supernatant samples were also analyzed with LabChip (PerkinElmer, Waltham, MA, USA). For protein mixtures adsorption isotherms, loading and supernatant samples were analyzed with the LabChip to get protein concentration values (see Section 2.4.2) and each dataset was repeated twice.

### 2.3. Mass Balance Equations

Adsorption isotherms were determined with a batch method in high-throughput mode. By using this methodology, a certain amount of protein is put in contact with a known amount of equilibrated resin until the equilibrium is reached in isotherm conditions at 25 °C. The total mass balance (Equation (1)), applied per each well, allows us to determine the value of adsorbed protein concentration. The values of loaded and unbound protein concentrations were measured.

(1) $$V_{medium}\cdot {q=V}_{liquid}\left(c_{0}\;{-\;c}_{unbound}\right)$$

(2) $$c_{0}=\frac{c_{sample}\cdot V_{sample}}{V_{sample}{\;+\;V}_{r}}$$

(3) $$V_{liquid}{=V}_{sample}{\;+\;V}_{r}$$

(4) $$V_{r}{=V}_{medium}\cdot 0.6+6\;\mu L$$

*V_medium_* is the apparent volume of the adsorbent particles, *V_sample_* is the volume of loaded protein solution and *V_liquid_* is the total liquid phase volume in each well. We must consider that a certain amount of liquid is retained from the solid phase and the membrane at the bottom of the filter plates. For this reason, the protein solution is slightly diluted when loaded into the well and this must be considered in the mass balance. *V_liquid_* includes the retained volume *V_r_*, which has been determined experimentally in a previous work [11] and is equal to the volume retained by the adsorbent phase (60% of the adsorbent phase volume) plus the liquid volume retained in the filter membrane (6 μL) (Equations (2)–(4)). *c_sample_* is the loaded protein concentration while *c*_0_ is the corrected protein concentration in the well. *q* is the adsorbed protein concentration in equilibrium with *c_unbound_*, the unbound protein concentration.

### 2.4. Analysis

#### 2.4.1. UV-Vis Spectrophotometer

Regarding the determination of mono-component isotherms, analysis of loading and supernatant samples was performed at 280 nm to obtain absorbance values. It was observed that salt buffers do not produce interference at this wavelength. Concentration values were obtained by calibration curves from the absorbance values of loadings and supernatants.

#### 2.4.2. Microfluidic Capillary Electrophoresis

The LabChip GXII Touch protein characterization system provides separation of different species via capillary electrophoresis and quantification via fluorescence analysis with reduced and non-reduced proteins (this second method was exploited in this work). The LabChip system analyzes each sample in 42 s and determines the concentration and sizing (via calibration curves) and purity. A very small amount of sample is needed for the analysis (only 2 μL) and this is entirely consistent with the high-throughput methodologies and goals.

The LabChip and samples were prepared according to the Protein Express Assay Quick Guide [12]. Filter tubes, ladder and buffer tubes were provided together with the reagent kit. NuPAGE Sample Reducing Agent from Thermo Scientific was used to reduce samples before analysis. HardShell PCR Plate with 96 wells from Perkin Elmer were used to prepare and analyze samples with the LabChip System. The analysis can be performed in two ways depending on the amount of protein in the sample. With the standard sensitivity mode, 2 μL of protein solution are denatured at 100 °C for five minutes with 7 μL of reducing solution and, after denaturation, 35 μL of water are pipetted into the wells to stop the reaction. Using the high sensitivity mode, the volume of the protein solution in the sample is higher (7 μL) but the total volume in the well is the same as the standard sensitivity mode. This second analysis modality allows the analysis of samples that have concentrations that would be below the limit of detection when prepared with the standard sensitivity mode. The choice of analysis mode can be made knowing that the limit of detection of the instruments is 5 μg/mL. We used the standard sensitivity mode since pure proteins were used with known concentrations. Furthermore, lysozyme has high adsorption in fluorescence; if the sample is too concentrated the peak in the electropherogram is too high and it goes towards saturation of the sensor, thus the analysis fails.

### 2.5. Procedure Evaluation

#### 2.5.1. Precision Evaluation

High-throughput methods are very efficient because a large number of parameters can be investigated in parallel, and a small amount of sample and chemicals are needed, but the procedure for filter plate preparation is quite complex and has a lot of passages; most of the experimental errors that can be committed cannot be quantified a priori because they mostly depend on operator precision (pipetting resin slurry or buffers) and conditions that cannot be easily controlled (homogeneity of the resin slurry, possible protein aggregation in the wells); therefore, it is useful to evaluate the overall error statistically.

In this work, the precision of the high-throughput method was evaluated to assess the experimental procedure described in Section 2.2.

The standard deviation was evaluated for a limited set of conditions for single and multi-component adsorption isotherms. In the case of single-component isotherms, experimental tests with filter plates were performed to investigate two different conditions per each protein or mixture: 10 mg/mL of protein concentration without salt in the buffer and 10 mg/mL protein concentration with 1 mol/L of salt in the buffer. Each condition as described above, was performed in 32 wells in the filter plate.

In the condition without salt in the buffer, no (or very low) adsorption occurred in the wells for the three proteins, so this condition was chosen to evaluate the experimental error resulting only from manipulation, excluding adsorption. In the case of 1 mol/L of ammonium sulfate in the buffer, the adsorption of the protein to the solid is higher [9], hence, variability in the adsorption behavior of the protein per each well can be evaluated along with the experimental procedure.

In the case of protein mixtures, the condition investigated for the reproducibility study was 50%BSA–50%CRM_197_ using a total protein concentration of 5 mg/mL with 0.5 mol/L of ammonium sulfate in the buffer. The condition described here was investigated in 30 wells of the filter plate. This combination of lower protein concentration with lower salt concentration, with respect to the single component tests, results in basically the same effect, from the adsorption point of view, of higher salt and protein concentrations. The salt concentration was chosen in such a way that the protein was almost equally distributed between the solid and liquid phases, and the latter had a concentration within the linear range of fluorescence analysis (0–2 mg/mL). This choice was made to avoid diluting the samples that must be analyzed as this is an operation that would increase the manipulation of the samples and therefore the variability. We must note that the protein concentration used for the protein mixture reproducibility investigation was four times lower than the single component test. For this reason, the salt concentration used in this case was lower (0.5 mol/L); if a higher concentration was used, the adsorption would be too high, and no peaks would be detected in the flowthrough.

The supernatant of the filter plate was analyzed with a UV-Vis spectrophotometer in the case of a single-component system, and with the LabChip in the case of binary mixtures.

The standard deviation for the obtained values of absorbance, in the UV-Vis analysis, and peak area, in the case of the LabChip analysis, was calculated together with the %RSD and referred to the average value. In both cases, the concentration values were obtained with calibration curves from absorbance and peak area values; standard deviation and %RSD were also calculated for the concentration data that was obtained. These last values of %RSD, and standard deviation also include the error committed by using the calibration curve to obtain concentration values.

Values of adsorbed protein concentration are affected, as mentioned above, by a series of experimental errors that occur during preparation and analysis. Some of these errors are not easily determinable. Moreover, *q* values are not directly measured but are calculated with the mass balance from values of *c*_0_ and *c_unbound_*. For these reasons, the error bars applied to adsorbed protein concentration values are represented by the %RSD obtained from the tests described above, considering the condition of adsorption in the wells.

In the case of UV-Vis analysis for isotherm determination, for the whole set of data, the semi-dispersion, calculated as the semi-difference between the maximum and minimum concentration value, was also considered.

#### 2.5.2. Evaluation of the Analysis Error

Concentrations of loading and supernatant were measured with UV-Vis spectrophotometer for single-component isotherms, and with LabChip for multi-component isotherms. Absorbance analysis and capillary electrophoresis are different analytical techniques, but the procedure steps are similar including: preparation of samples, analysis, and the use of calibration curve to obtain concentration values. Thus, the same approach was used to evaluate the error affecting the measured concentration values for both analytical methods.

The measure is mainly affected by the equipment variability and the error that occurs in using the calibration curve to convert absorbance and peak area values into concentration values, as discussed in Section 3.1.2.

The same sample was analyzed a certain number of times. In the case of absorbance analysis, it was performed on 16 samples of BSA at 1 mg/mL. As done in the previous section, the standard deviation and %RSDs are calculated for absorbance values. For each protein, then, the calibration curve error was calculated as the average of the relative errors of each point of the curve.

The final error bars applied to the *c*_0_ and *c_unbound_* concentration values consider the sum of the equipment measurement variability and calibration curve error in the case of the UV-Vis spectrophotometer.

The same study was performed on 1 mg/mL BSA samples, analyzed with the LabChip. In this case the standard deviation and %RSD were calculated for peak area and concentration data. The calibration curve error was calculated also for LabChip analysis, in the same way described for the UV-Vis case.

Additionally, in the case of LabChip, we wanted to determine the measurement error, excluding the variability resulting from the preparation of the samples, to assess the precision of the instrument only, and to determine how much the preparation of samples affects the measurement. The test was carried out by preparing a larger amount of sample, according to the procedure described in Section 2.4.2 and dispensing it into ten wells. This should remove some of the variability introduced with the preparation and denaturation of ten samples in ten different wells.

In the case of the electrophoresis analysis, the error bars also consider the sum of equipment variability (with manipulation) and absolute error of calibration curve.

Since the capillary electrophoresis is not such an established method for the determination of multi-component adsorption isotherms as absorbance analysis with UV-Vis spectrophotometer is for single-component isotherms determination, an accuracy validation was also performed for the LabChip.

A BSA standard sample (BSA standard at 2 ± 0.03 mg/mL calibrated by direct comparison to purified BSA from the National Institute of Standard and Technology, purchased from Thermo Fisher (Waltham, MA, USA) was diluted and analyzed five times with the LabChip and the concentration value so obtained was compared with the value provided by the manufacturer. The BSA sample was diluted 1:1 to reach a concentration of 1 mg/mL since the linearity in fluorescence analysis between concentration and peak area is, for most of the proteins, guaranteed under a concentration of 2 mg/mL. The error made for the dilution of the sample was considered negligible.

#### 2.5.3. Separation Efficiency Evaluation

The Protein Express Assay LabChip separates proteins by their molecular weight. A 90%CRM_197_-10%BSA and 10%CRM_197_-90%BSA samples were analyzed in order to verify if the separation occurred correctly. BSA and CRM_197_ were used for the test because they have similar molecular weights (66.4 kDa and 58.8 kDa, respectively).

## 3. Results and Discussion

### 3.1. Procedure Validation

#### 3.1.1. Precision Investigation

For the three proteins in a single-component system, the reproducibility of the high-throughput method was evaluated as explained in Section 2.5.1.

The results of the experiments are shown in Table 1 and Table 2.

The standard deviation of no/low-adsorption absorbance and concentration values were generally slightly lower than the case of 1 mol/L of salt in the buffer. While calculating the standard deviation, outliers were identified with the Tukey criteria [13] and excluded.

The difference between standard deviation and %RSD values of absorbance and concentration in the two different adsorption conditions shows how the adsorption in plates is affected by random variations that cannot be directly quantifiable.

The semi-dispersion calculated for the UV-Vis set of data was found to be between 1% and 15% approximately. The semi-dispersion values were generally slightly higher as the adsorbed concentration increased. This behavior is consistent with what was found previously: adsorption increases variability in the procedure.

In the case of protein mixtures, the results of the test in mild adsorption conditions are shown in Table 3. In this case, the variables investigated were the peak area of the resulting electropherogram and the related concentration obtained with the calibration curve.

**Figure 1:**
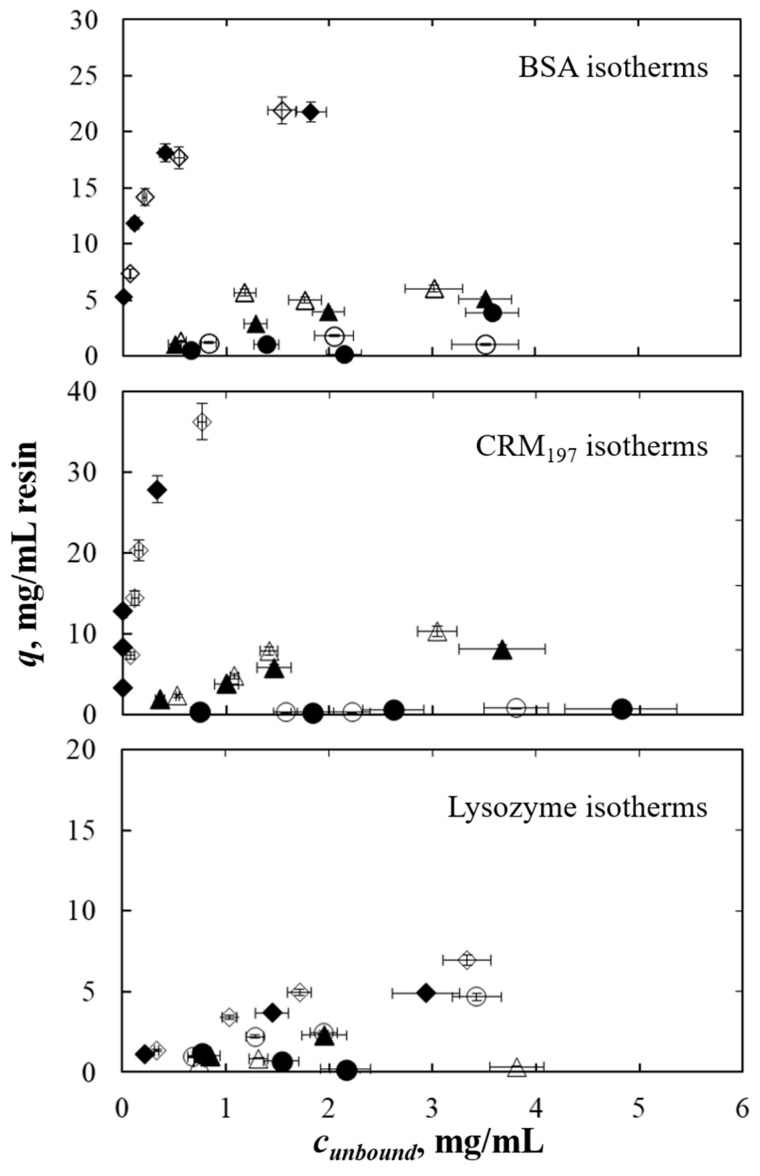
Comparison of adsorption isotherms obtained with UV-Vis spectrophotometer and LabChip for the BSA, CRM_197_ and lysozyme when investigated as a single-component at different salt concentrations. For each protein, full symbols refer to LabChip analysis, while empty symbols refer to UV-Vis spectrophotometer analysis. Circles, triangles and diamonds refer to isotherms obtained at 0, 0.5 and 1 mol/L of ammonium sulfate, respectively.

The value of %RSD calculated for the samples was considered and applied to *q* concentration values (*y*-axis) for both UV-Vis and microfluidic capillary electrophoresis analysis (see Figure 1). In the case of protein mixtures, the semi-dispersion calculated for experimental data was between 2 and 19% in the mixtures that were experimentally investigated, BSA–CRM_197_ and CRM_197_–lysozyme, while the mixture of BSA–lysozyme gave abnormal results that are discussed in Section 3.3. For this reason, and since semi-dispersion values were coherent between the different mixtures, the lysozyme variability was not experimentally investigated, but assumed as the average of BSA and CRM_197_ %RSDs.

%RSD, and then precision, has been reported as approximately 1.6–2.1% in a previous work [14], but in that case a robotic platform was used. Furthermore, the precision of adsorbed protein concentration was found to be less than 3.6% in another work [7] where multivariate analysis of spectra had been exploited for analysis. In this last case, a robotic liquid handler was also used. Results reported here were higher, but it must be noted that all the operations described were performed manually by the operator. Moreover, it has been demonstrated in Osberghaus et al. [14] that the precision of the procedure and analysis increases with the training of the experimenter on the device. The manipulation factor mainly affects the results.

#### 3.1.2. Evaluation of the Analysis Error

The UV-Vis spectrophotometer measurement variability was investigated as explained in Section 2.5.2. The %RSD turned out to be 4.06% with a standard deviation of 0.0018 AU.

The relative error was calculated for all the curve building points. The final calibration line error considered was the average value of all the relative errors of the line building points. The average relative errors and correlation coefficients of the UV-Vis calibration curves of BSA, CRM_197_ and lysozyme are reported in Table 4. The sum of the UV-Vis spectrophotometer measurement error and calibration line error was applied to *c_unbound_* values (*x*-axis) for single-component adsorption isotherms.

In the case of microfluidic capillary electrophoresis, the measurement variability was calculated on the peak area obtained from the electropherograms of the samples. In this case the %RSD was 6.35%.

The calibration curve error was calculated for LabChip analysis with the same procedure used for UV-Vis analysis. The calibration curve average relative errors calculated for BSA, CRM_197_ and lysozyme are reported in Table 5. The sum of %RSD and calibration curve absolute error was applied to the *c_unbound_* concentration values (*x*-axis) in multi-component isotherms (see Figure 1).

To determine how the preparation of the samples affects variability in microfluidic electrophoresis, a larger amount of sample (BSA 1 mg/mL) was prepared as described in Section 2.5.2 and dispensed into ten wells to exclude sample preparation variability. The %RSD in this case was 1.09%, which is lower than the previous case. This difference shows how the manipulation necessary to prepare samples, affects the measurement error more than equipment variability.

Five samples of standard BSA at 1 mg/mL were analyzed with the LabChip as described in Section 2.5.2. Concentration values of the samples were obtained using the calibration curve and standard deviation and %RSD were calculated on concentration values. As previously explained, the standard sample of BSA 2 mg/mL was diluted 1:1 in order to remain in the linear range of fluorescence analysis. The average value of the concentration of the samples was 1.070 mg/mL, and the semi-dispersion of the five concentration values was 0.071 mg/mL. The accuracy, calculated with Equation (5) (*c_av_* and *c_th_* are the average and theoretical concentration, respectively), was 7.04%; this value also considers the variability resulting from the manipulation of each sample, which is the main cause of error in the procedure. It can be noted that in the work of Field et al. [7], the accuracy of the PLS method was within 5%, which is not far from the accuracy that was determined for this application of capillary electrophoresis. The accuracy study was done at a unique point of the calibration curve in the middle of the working range of concentrations (0–2 mg/mL). We assume that the results so obtained are valid for all the concentrations belonging to the calibration curve.

(5) $$Accuracy=\frac{c_{av}{\;-\;c}_{th}}{c_{th}}\times 100$$

#### 3.1.3. Separation Efficiency Investigation

**Figure 2:**
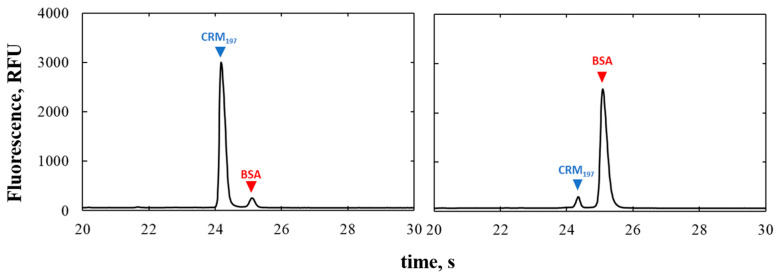
Electropherograms of binary mixtures of 90%CRM_197_-10%BSA (**Sample 1**), on the left, and 10%CRM_197–_90%BSA (**Sample 2**), on the right.

Electropherograms obtained from the analysis of 90%CRM_197_-10%BSA (Sample 1) and 10%CRM_197–_90%BSA (Sample 2) mixtures are shown in Figure 2. As written above, BSA and CRM_197_ have similar molecular weights, nevertheless, the peak resolution was high, the two peaks turned out to be well separated. In both cases, the protein present in smaller quantities was adequately separated. Values of protein fractions were obtained from LabChip analysis (Table 6). The experimental protein fractions were reasonably close to the theoretic values, considering errors that could occur in diluting a single protein solution to reach the desired concentration and mixing them in the desired ratio, in addition to the sample preparation required for analysis. Indeed, as demonstrated in Section 3.1.2, manipulation of the sample mainly affects the measurement. Thus, we can say that no significant amounts of protein were mixed with each other during separation, and the peaks were representative of the individual separated proteins. The difference between theoretical and experimental fraction values is assumed to be due to sample handling.

### 3.2. Single-Component Isotherm Comparison

Single-component isotherms obtained with UV-Vis analysis were consistent with those obtained with LabChip analysis (Figure 1). It can be seen that BSA, CRM_197_ and lysozyme adsorption isotherms obtained with the two different methods were basically the same, even considering that the preparation of the samples in the case of LabChip is more complex (see Section 2.4.2) than UV-Vis analysis.

### 3.3. Multi-Component Adsorption Isotherms

Binary mixtures adsorption behavior was investigated. Mixtures were prepared according to the procedure in Section 2.2 and isotherms were obtained by applying mass balance (Equation (1)) to the single protein concentrations.

**Figure 3:**
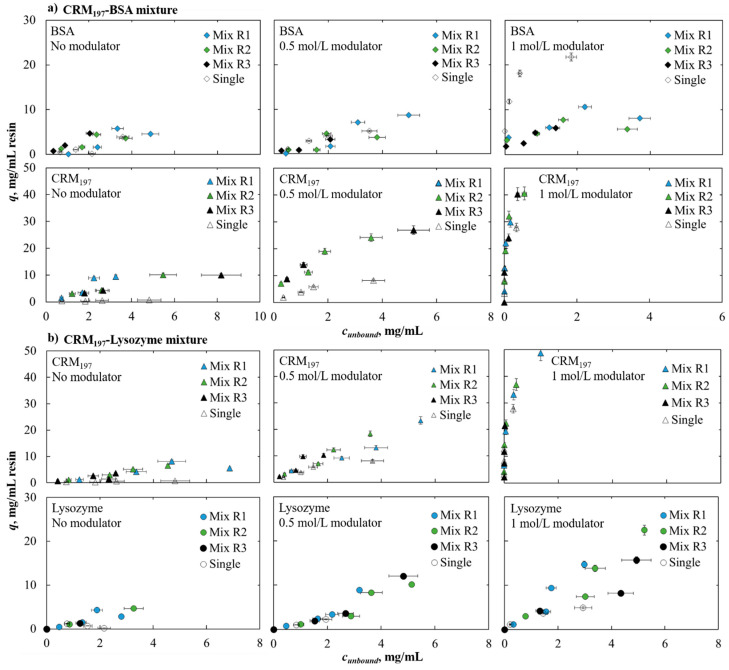
Adsorption isotherm comparison for binary mixtures: section (**a**) shows the mixture BSA–CRM_197_, section (**b**) shows the mixture CRM_197_–lysozyme. Each graph presents data for a fixed ammonium sulfate concentration (0, 0.5 and 1 mol/L from left to the right). In each graph, the empty symbols labelled as “Single” represent the adsorption isotherm of the protein in single-component case (see Figure 1), “Mix R1” is the protein ratio 70–30%, “Mix R2” is the protein ratio 50–50%, “Mix R3” is the protein ratio 30–70% considering that in all the ratios, the first percentage is relative to the protein shown in the upper graphs of the section and the second percentage is relative to the protein in the lower graphs of the section.

In Figure 3a, the comparison of single and multi-component isotherm is shown for BSA and CRM_197_. With these two proteins, we noticed that in the case of 0 and 0.5 mol/L of ammonium sulfate in the buffer, the mutual adsorption behavior did not change when the protein was in the mixture. When the concentration of the salt was 1 mol/L, the BSA adsorption isotherm, when in a mixture with CRM_197_, was lower than the single-component isotherm. CRM_197_ could affect BSA adsorption by reducing its binding capacity.

The isotherms for the CRM_197_–lysozyme mixture are shown in Figure 3b. In 0 and 0.5 mol/L of ammonium sulfate in the buffer, multi-component isotherms followed the single-component isotherms behavior; in these conditions, CRM_197_ and lysozyme showed low adsorption. With 1 mol/L of salt in the buffer, the adsorption for both proteins was higher, and also in this case, both followed their single-component behavior.

It can be noticed that changing the ratio of proteins did not affect the adsorption behavior of the proteins in both cases. The concentration of the modulator affects the entity of the adsorption. It is known that a higher salt concentration results in higher protein adsorption, since the presence of the salt in the liquid phase reduces the solubility of the protein, which, in this way, tends to interact with the active sites in the solid phase.

The binary mixture of BSA and lysozyme was also investigated, but in this case the tests gave abnormal results. In particular, the plate with BSA–lysozyme mixture was tested three times and concentration values obtained from the analysis were variable; the values were in some case too high or too low and in any case they were inconsistent. Furthermore, the single component adsorption isotherms obtained (on the same plate) with the LabChip were not consistent with the adsorption isotherms obtained with the UV-Vis analysis. This did not happen in the case of the other two mixtures, as previously shown. This problem could be due to some sort of interference of this mixture with the gel electrophoresis, or to the difference in surface hydrophobicity and intramolecular forces of the two proteins [15]. Further studies are required to clarify this behavior.

**Figure 4:**
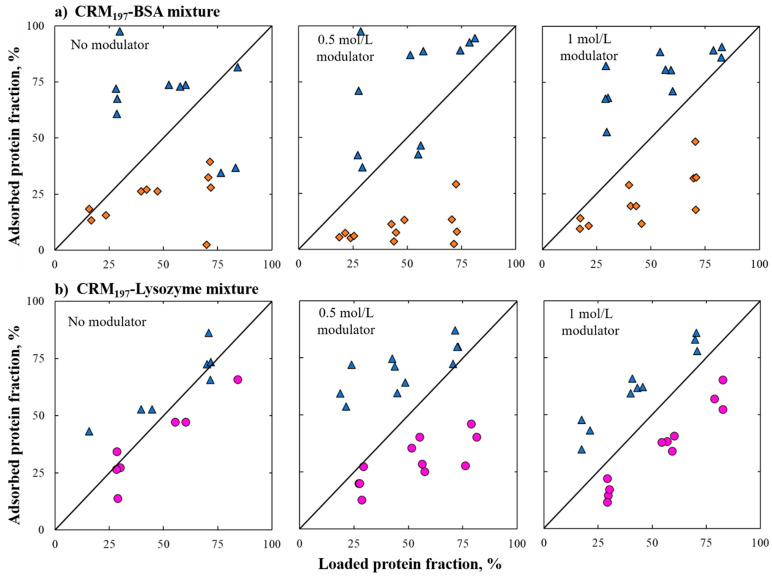
Parity plots describe the relationship between adsorbed and loaded protein fractions at different salt concentrations. Blue triangles represent CRM_197_ fractions, pink circles represent lysozyme fractions and orange diamonds represent BSA fractions. Section (**a**) BSA–CRM_197_ and section (**b**) CRM_197_–lysozyme mixture.

The different adsorption behavior of proteins could also be evidenced when reporting adsorption data on parity plots (Figure 4). The experimental protein ratios were reported in parity plots of loaded protein fractions (*x*-axis) and adsorbed protein fractions (*y*-axis). From the parity plots, it can be noted that protein fractions are always close to the bisector in the case of the CRM_197_–lysozyme mixture, and for this reason, the parity plots confirmed that protein adsorption behavior did not change when they were in a mixture compared to what was observed in a single-component system. In the case of the BSA–CRM_197_ mixture, it can be noticed that, especially in the case of 1 mol/L of ammonium sulfate, for BSA the adsorbed fraction was generally lower than the loaded fraction. Thus, the opposite happened to the CRM_197_, for which the adsorbed fraction was higher than the loaded fraction. In these graphs, it can be noticed that the data are stratified and grouped according to the different protein ratios used. Indeed, the graphs show the correspondence between the data series and the protein ratios used. The higher the loaded protein concentration, the higher the protein fraction adsorbed to the solid. Additionally, Figure 4 highlights the impact of salt concentration on the mixture separation efficiency.

## 4. Conclusions

Multi-component isotherm determination is crucial to better understand chromatographic purification processes. Adsorption isotherms determination allows us to more closely investigate the adsorption mechanisms of chromatography, in which the separation of components is mainly affected by these mechanisms. The knowledge obtained on the adsorption behavior can be used to model the separation that occurs in a chromatographic system, and to speed up and optimize the development of the purification process. The quantification of protein concentrations in mixtures has been a bottleneck for multi-component isotherm determination.

In this work, we proposed a rapid (42 s per sample) and reliable method for protein quantification in multi-component isotherm determination. Microfluidic capillary electrophoresis in a high-throughput platform was used to quantify protein concentrations to build multi-component adsorption isotherms of some proteins to investigate their interaction with hydrophobic resin. By using this equipment to analyze protein mixtures, the analytical process times were greatly reduced. When a high-throughput method is used to determine isotherms, a high number of samples need to be analyzed. Using traditional analytical methods, such as HPLC or UPLC, is not suitable because they take too long, thus losing the high-throughput advantages, despite having high precision. The microfluidic capillary electrophoresis is a fast and easy method to quantify protein mixtures in high-throughput mode for adsorption isotherm determination. Furthermore, this method is more accurate than most of colorimetric assays (e.g., BCA or Lowry protein assays) used for routine analysis in the process development of a biopharmaceutical.

High-throughput methodologies are a powerful tool for process screening. On the other hand, since high-throughput experimentations require a high level of experimental effort, the experimental error that can occur during manipulation and analysis must be evaluated. Precision and accuracy of the equipment were evaluated in order to assess this methodology for isotherm determination.

When capillary electrophoresis was compared to UV-Vis absorbance analysis, the single-component isotherms obtained with the two methods were consistent. Microfluidic capillary electrophoresis was reliable in the determination of single-component adsorption isotherms.

The precision of the high-throughput procedure was investigated by performing tests in low and mild adsorption. The %RSD was quite low and demonstrates how high-throughput methodologies are reliable, even though the experimental procedure and analysis are performed manually.

Moreover, we demonstrated that measurement error, in the case of capillary electrophoresis analysis, is mainly affected by manipulation for sample preparation. The measurement variability, excluding manipulation, was around 1%, which is a very low equipment variability.

The separation efficiency of the microfluidic capillary electrophoresis platform was also investigated, and we showed that even though the proteins present in the mixtures have similar molecular weight, they were well separated and the resolution of the peaks in the electropherograms was high.

A binary mixture of the proteins mentioned above were then investigated and we showed that in the case of the CRM_197_–lysozyme mixture, the two proteins follow their single-component behavior; in the case of the BSA–CRM_197_ mixture at higher salt concentration, BSA adsorption is lower than in the single-component system. Furthermore, different ratios of proteins in mixtures do not affect adsorption behavior, in the conditions investigated here.

Adsorption dynamics in hydrophobic interaction chromatography are still not well understood. Several models have been developed to describe adsorption equilibrium and kinetics with different constraints proposed [16,17,18]. Most of the models developed are theoretically supported and adapted for multi-component adsorption isotherms. The application of the capillary electrophoresis described here could help in developing or improving models to describe adsorption equilibrium in hydrophobic interaction chromatography, thus filling the gaps in the data on mixtures in the literature.

Apart from being of interest for purification processes, multi-component adsorption isotherms determination of protein is a method that evidences interactions and may help to clarify mechanisms and small differences between different proteins, as well as the influence of differences in surface hydrophobicity and surface charge distribution.

This work aims to demonstrate how microfluidic capillary electrophoresis is a powerful and reliable way to analyze protein mixtures for quantification. This method, in a high-throughput platform, coupled with the well-known 96-well filter plates procedure, determines multi-component isotherms in a very fast and easy way, obtaining robust and accurate results.

The method described here is not limited to multi-component isotherm investigation, but it can be applied to every kind of high-throughput process screening. It can especially be applied in the scale up phase of the process development of a biopharmaceutical product where the protein quantification of a large number of samples is needed, for example, for robocolumn process screening or even at larger scale.

## Figures and Tables

**Table 1:** Average value, standard deviation and %RSD for the proteins when analyzed with UV-Vis spectrophotometer in low adsorption conditions (without ammonium sulfate).

		BSA	CRM197	Lysozyme
**Absorbance, AU**	Average	0.274	1.850	0.964
	Standard Deviation	0.011	0.050	0.044
**Absorbance %RSD**		3.83	2.69	4.57
**Concentration, mg/mL**	Average	6.83	9.09	7.25
	Standard Deviation	0.29	0.25	0.35
**Concentration %RSD**		4.22	2.76	4.81

**Table 2:** Average value, standard deviation and %RSD for the proteins when analyzed with UV-Vis spectrophotometer in adsorption conditions (1 mol/L ammonium sulfate).

		BSA	CRM_197_	Lysozyme
**Absorbance, AU**	Average	0.458	0.756	2.48
	Standard Deviation	0.022	0.045	0.11
**Absorbance %RSD**		3.83	4.74	5.89
**Concentration, mg/mL**	Average	4.61	3.56	7.19
	Standard Deviation	0.25	0.23	0.31
**Concentration %RSD**		4.22	5.42	6.29

**Table 3:** Average value, standard deviation and %RSD for BSA and CRM_197_ when analyzed with LabChip in binary mixture and adsorption conditions (0.5 mol/L ammonium sulfate).

		BSA	CRM_197_
**Peak area, RFU’s**	Average	2300	1140
	Standard Deviation	100	60
**Peak area %RSD**		4.43	5.44
**Concentration, mg/mL**	Average	1.774	0.961
	Standard Deviation	0.074	0.057
**Concentration %RSD**		4.18	5.92

**Table 4:** Correlation coefficients and average relative errors of the UV-Vis calibration curves used for quantification of BSA, CRM_197_ and lysozyme.

	Calibration Line Correlation Coefficient R^2^	Average Relative Error, %
**BSA**	0.9989	5.15
**CRM_197_**	0.9997	2.54
**Lysozyme**	0.9998	2.80

**Table 5:** Correlation coefficients and average relative errors of the capillary electrophoresis calibration curves used for quantification of BSA, CRM_197_ and lysozyme.

	Calibration Line Correlation Coefficient R^2^	Average Relative Error, %
**BSA**	0.9996	1.74
**CRM_197_**	0.9957	4.98
**Lysozyme**	0.9997	4.72

**Table 6:** Protein fraction obtained with LabChip analysis on the BSA–CRM_197_ mixture.

Sample	CRM_197_ Fraction, %	BSA Fraction, %
Sample 1	92.88	6.88
Sample 2	5.75	94.25

## Data Availability

All data available are reported in the article.
